# Plasma Pharmacokinetics of Cannabidiol Following Oral Administration of Cannabidiol Oil to Dairy Calves

**DOI:** 10.3389/fvets.2022.789495

**Published:** 2022-01-24

**Authors:** Kelsey Meyer, Kristen Hayman, James Baumgartner, Patrick J. Gorden

**Affiliations:** ^1^Department of Veterinary Diagnostic and Production Animal Medicine, Iowa State University College of Veterinary Medicine, Ames, IA, United States; ^2^Panacea Life Sciences, Golden, CO, United States

**Keywords:** cannabidiol, CBD oil, pharmacokinetics, cannabis, calves

## Abstract

Cannabidiol (CBD), the non-psychotropic component of cannabis, has drawn increased interest amongst some medical professionals for its potential therapeutic effects. Human and canine work has been done to describe CBD where it is already widely used, however, little is known about the effects of CBD in livestock species. The purpose of this descriptive study was to determine the pharmacokinetics (PK) of CBD in calves after a single oral exposure to CBD oil. Seven male Holstein calves received a single oral dose of 25 mg/mL CBD oil to achieve 5 mg/kg dose of CBD. Blood samples were collected for 48 (h) after dosing. The CBD geometric mean maximum concentration of 0.05 ug/mL was reached 7.5 h after administration. The geometric mean half-life was 23.02 h. Cannabidiol administered orally to cattle is slowly absorbed and has an extended elimination half-life compared to other species.

## Introduction

Cannabinoids have been used for thousands of years, recently attracting widespread attention due to the promising therapeutic benefits in human medicine. As a result, therapeutic consideration has extended to veterinary medicine, initially in companion animal species but quickly progressing to livestock production.

*Cannabis sativa* contains over 500 compounds, 150 of which are categorized as cannabinoids. The two main components of Cannabis are delta-9-tetrahydrocannabinol (THC) and cannabidiol (CBD). Much of the research on cannabinoids concentrates on THC, the psychotropic constituent, due to the intoxication effects in humans. While THC has been the focus, there has been increasing interest in CBD, the non-psychotropic component of Cannabis, due to its potential analgesic, anti-inflammatory, antioxidant, anxiolytic, anticonvulsant, and cytotoxic effects to cancer cell lines.

Currently, the pharmacokinetic (PK) properties of CBD have been reported in humans and dogs. The studies completed on dogs concluded that oral administration resulted in higher bioavailability compared to transdermal administration (1) with a plasma half-life reported to be 2–4 (h) (1, 2). Additionally, CBD has been investigated for its range of therapeutic properties in humans, lab rodents, dogs, and horses. In horses, CBD has shown positive effects on reducing chronic pain and anxiety (3). Gamble et al. showed increased comfort and activity in dogs diagnosed with osteoarthritis after treatment with CBD (1). From the limited studies completed, CBD seems to be therapeutic without significant adverse effects (1, 3–6).

Therapeutic effects and PK of CBD have not been published in production animal species, such as cattle. However, recently a PK study by Kleinhenz et al. (7) investigated cannabinoids, specifically cannabidiolic acid (CBDA) the precursor to CBD, following oral administration of industrial hemp. The objective of this study was to describe the PK of cannabidiol administered orally as an oil formulation to cattle.

## Methods and Materials

### Animals

Iowa State University's Institutional Animal Use and Care Committee approved the research protocol prior to commencement of trial procedures (protocol number 20–102). This study was completed at the Iowa State University Dairy Farm. Seven 19.43 ± 5.29 day-old Holstein bull calves weighing 48.61 ± 4.72 kg at the time of the study were used. The calves were purchased from a commercial farm and moved to the university farm, where they were acclimated for 10 days prior to study initiation. During the course of the trial, the calves were housed in outdoor hutches. Calf housing and management met or exceeded the recommendations listed in the Guide for Care and Use of Agricultural Animals in Research and Teaching (8). Calves were fed 3 quarts of milk replacer twice daily at 7:00 and 17:00 h. Following milk consumption, warm water was immediately offered *via* the bottle. Free choice water and a commercial calf starter available to calves throughout the trial. Demeanor, hydration status, and appetite were assessed daily to monitor calf health. Cattle used for this study were not allowed to enter the food chain as CBD is not an approved drug by the US Food and Drug Administration.

### Experimental Design and Blood Collection

Catheters (16G × 2,” Jorgenson Laboratories, Loveland, CO) were placed in one jugular vein and the animals were weighed on the day prior to cannabidiol administration. Cannabidiol was administered orally at 5 mg/kg (cannabidiol oil formulated at 25 mg/ml, Panacea Life Science, Golden, CO) *via* a 20 mL syringe placed in the mouth and slowly dispensed while the calf suckled the syringe. Calves were allowed to suckle for 15 s post administration to prevent leakage of cannabidiol from the mouth.

Two 10-mL blood samples were collected in glass heparin tubes (Sodium Heparin^N^ BD Vacutainer, BD Biosciences, Franklin Lakes, NJ) *via* the jugular catheter at several time points to be used for plasma CBD analysis. The first sample was collected 10 min prior to the administration of CBD oil and served as a baseline value (T0 h). Blood samples were then collected at 0.25, 0.5, 1, 2, 4, 6, 8, 12, 18, 24, 30, 36, 42, and 48 h after CBD oil administration. Blood was immediately placed on ice and transported to the laboratory within 1 h of sampling and centrifuged for 20 min at 2,700 × g at 4°C. Plasma was harvested and stored at −80°C until analyzed for drug concentration.

### Plasma CBD Concentration Analysis

Plasma concentrations of CBD were determined using ultra high-pressure liquid chromatography (UHPLC) with mass spectrometry detection after liquid-liquid extraction into methyl tert-butyl ether (MTBE). The UHPLC system consisted of a pump, column compartment, and autosampler (Thermo Scientific, San Jose, CA, USA) coupled to an Orbitrap mass spectrometer (Q Exactive Focus, Thermo Scientific, San Jose, CA, USA). Bovine plasma samples were thawed and centrifuged (2,500 × g) prior to analysis.

Certified reference standard (cannabidiol at 1.00 mg/mL and d3-cannabidiol at 100 ug/mL) diluted in ethanol were obtained (Cerilliant, Red Rock, TX). Twelve calibration spikes covering the concentration range of 0.20–2,000 ng/mL and 4 quality control (QC) samples at 1.5, 15, 150, and 1,500 ng/mL prepared in blank bovine plasma were analyzed with each set of samples. Plasma samples, plasma spikes, plasma QC, and bovine plasma blanks (200 μL) were extracted with 5 mL of MTBE on a multitube vortexer for 5 min at 2,000 rpm. Prior to the addition of MTBE, 1 mL of water was added to the samples followed by 20 μL of an internal standard solution of cannabidiol-d3 at 10.0 ng/μL. After removal from the multitube vortexer, the samples were centrifuged for 10 min at 2,500 × g and the organic layer transferred by pipette to 16 x 100 mm glass culture tubes. The MTBE was evaporated at 30°C with a flow of nitrogen in a Turbovap. The contents were reconstituted with 200 μL of 50% acetonitrile in water. The samples were transferred to autosampler vials fitted with a glass insert and centrifuged at 2,000 × g prior to analysis.

The analysis was performed with a 50 × 2.1 mm, 1.9 μm particle column (Hypersil Gold Vanquish, Thermo Scientific, San Jose, CA, USA) maintained at a temperature of 45°C and the autosampler at temperature of 10°C. The analysis used an injection volume of 3.0 μL. The mobile phases consisted of A: 0.1% formic acid in water and B: 0.1% formic acid in acetonitrile. The starting solvent composition was 50% B at a flow rate of 0.350 mL/min, which was increased linearly to 95% B in 3.75 min. The solvent composition was maintained at 95% B for 1 min prior to equilibration back to 50% B. The flow rate during this time period was 0.45 ml/min. Cannabidiol and cannabidiol-d3 eluted from the column at 3.21 ± 0.02 min. Parallel reaction monitoring in the positive electrospray ion mode was used for analyte detection with a spray voltage of 4.0 kV and an electrospray temperature of 225°C. A collision energy of 25 electron volts (eV) was used for fragmentation of all the CBD analytes within the collision cell. The precursor ions were determined by the instrument software from the molecular formulas. These were cannabidiol at a mass to charge ratio (m/z) of 315.232 and CBD-d3 at a m/z 318.251. Four fragment ions were used for quantitation of CBD, which were at 93.070, 135.117, 193.122, and 259.169 m/z, while ions at a m/z of 196.141 and 262.188 were characteristic of CBD-d3 fragmentation.

After a sequence of bovine plasma blanks, calibration spikes, QC's, and plasma samples were analyzed, the raw MS data was batch processed in commercial software (Xcalibur, Thermo Scientific, San Jose, CA, USA). Plasma concentrations of CBD in unknown samples were calculated by the software based on a weighted (1/X) linear fit calibration curve. The limit of quantitation (LOQ) of the analysis was 1.0 ng/mL with a limit of detection (LOD) of 0.2 ng mL. Calibration curves exhibited a correlation coefficient (*r*^2^) exceeding 0.999 across the concentration range. The 4 QC samples were within the ± 15% criteria of the nominal value, with the QC samples at concentrations above the lowest QC (>1.5 ng/mL) within ± 7.5% of the nominal value.

### Pharmacokinetic Analysis

A non-compartmental pharmacokinetic approach was used to analyze plasma drug concentration-time courses using a commercially available software program (PKanalix, version 2020R1, Lixoft SAS, Antony, France). Plasma concentration data was integrated using the linear up-log down approach and was weighted 1/y^2^ based on statistical moment theory, using the semi-logarithmic plots of CBD. Plasma concentration values below the LOQ before the T_max_ were entered as 0, while those after T_max_ were inferred to be LOQ/2. Peak plasma concentration (**C**_**max**_) and time to achieve measured peak concentration (**T**_**max**_) were determined directly from the plasma concentration plots. The terminal elimination rate constant (**λ_z_**) was calculated from the log-linear portion of terminal portion of the log plasma concentration-time curve using a linear regression technique. The elimination half-life (**T**_**1/2λ*z***_) was derived using the equation:


$$\begin{array}{l} T_{1/2\lambda \text{z}}\;=\;\frac{\ln\;2}{\lambda_{z}}. \end{array}$$

The area under the concentration time curve from T0 to T48 h (**AUC**_**last**_) was calculated using the linear-log trapezoidal method. To account for the total drug exposure to calves, area under the concentration time curve from the last measurement extrapolated to infinite time (**AUC**_**0−∞**_) and area under the moment curve to extrapolated to infinity **(AUMC**_**0−∞**_) were determined. Apparent volume of distribution (**V**_**z**_**/F**), apparent total body clearance (**CL/F**), and mean residence time (**MRT** = AUMC_0−∞_/AUC_0−∞_,) were also calculated.

### Statistical Analysis

Summary statistics for the individual PK parameters were determined using pharmacokinetic software (JMP Pro, version 14.3.0, SAS Institute, Cary, NC). A commercially available software program (Prism 8, Graph Pad, San Diego, CA) was used to create the CBD plasma vs. time semi-logarithmic concentration plots. Cannabidiol plasma concentration values below the LOQ before the T_max_ were entered as 0, while those after T_max_ were inferred to be LOQ/2.

## Results

**Figure 1:**
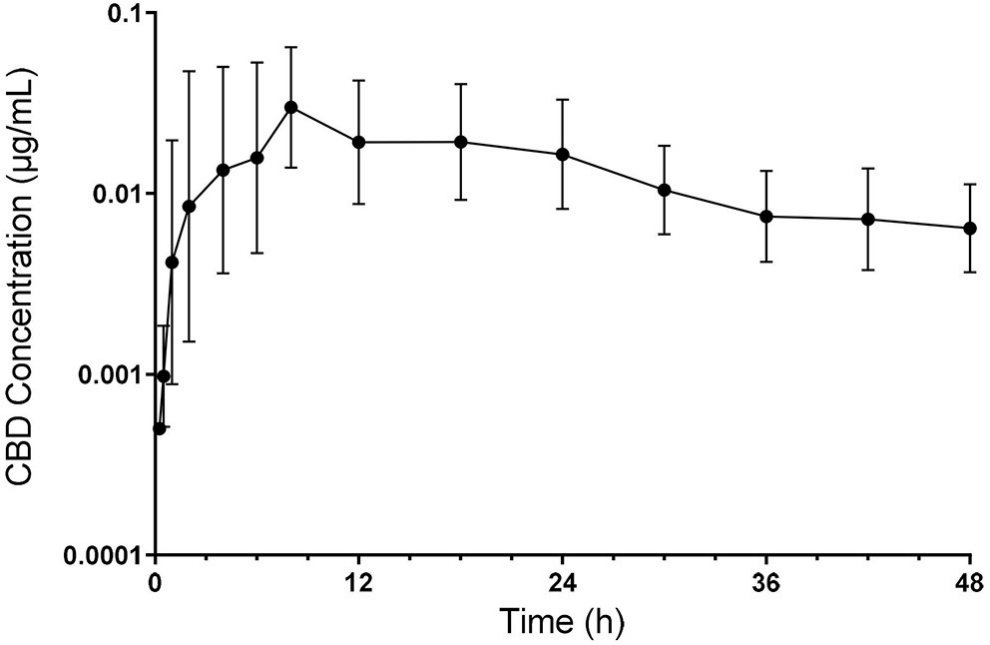
Semilogarithmic transformation of geometric mean plasma concentration (with 95% confidence interval) of CBD following oral administration at 5 mg/kg.

All calves enrolled completed the study with no observed adverse drug reactions or behavioral changes. Plasma CBD concentrations over time are shown in Figure 1. Pharmacokinetic parameters of CBD are summarized in Table 1. Geometric mean C_max_ plasma concentration of CBD was 0.05 ug/mL (range −0.01–0.09 ug/mL), which was reached 7.5 h (range −2–18 h) after oral administration. Area under the curve to the last time point (AUC_last_) geometric mean was 0.7 h^*^ug/mL (range −0.19–1.53 h^*^ug/mL) and the AUC_0−∞_ being 0.95 h^*^ug/mL with the extrapolated portion following the last time point representing 22.44% (range −10.62–48.64) of the value. The geometric mean of the mean residence time (MRT) was 35 h (range −25.25–67.55 h). Apparent volume distribution and apparent total body clearance geometric mean were determined to be 175.86 L/kg (range −58.36–526.92 L.kg) and 5.29 L/kg/h (range −2.92–20.77 L/kg/h), respectively. The geometric mean apparent half-life was 23.02 h (range −13.83–47.14 h).

**Table 1:** Individual animal and geometric mean pharmacokinetic parameters of cannabidol (CBD) following oral administration at 5 mg/kg.

		**Calf ID**									
**Parameter**	**Unit**	**6,495**	**6,521**	**6,523**	**6,551**	**6,553**	**6,586**	**6,589**	**Geometric mean**	**Median**	**Range**
AUC_0−∞_	h*ug/mL	1.30	1.71	0.24	1.04	0.92	1.26	1.07	0.95	1.07	0.24–1.71
AUC_%Extrap	%	16.32	10.62	23.63	19.75	20.48	48.64	35.54	22.44	20.48	10.62–48.64
AUC_last_	h*ug/mL	1.09	1.53	0.19	0.83	0.73	0.65	0.69	0.70	0.73	0.19–1.53
Cl_F	L/kg/h	3.86	2.92	20.77	4.85	5.5	3.99	4.68	5.29	4.68	2.92–20.77
C_max_	ug/mL	0.09	0.09	0.01	0.08	0.03	0.04	0.04	0.05	0.04	0.01–0.09
T_max_	h	6	18	8	2	12	8	8	7.5	8	2.0–18.0
Lambda_z	(h^−1^)	0.04	0.05	0.03	0.03	0.04	0.02	0.02	0.03	0.03	0.02–0.05
HL_Lambda_z	h	17.33	13.83	20.92	24.08	19.17	47.14	31.43	23.02	20.92	13.83–47.14
MRTINF	h	25.46	25.25	35.30	28.54	31.18	67.55	47.20	35.00	31.18	25.25–67.55
Vz_F	L/kg	96.53	58.36	626.92	168.45	152.06	271.26	211.97	175.86	168.45	58.36–626.92

## Discussion

This is the first study describing the pharmacokinetics of CBD in cattle after oral administration. Since the legalization of marijuana, companion animal and horse owners have been using cannabis to treat a multitude of medical conditions (9). The increased interest in used cannabis for its therapeutic effects has driven research into the safety and efficacy of the drug for all animal species.

Initial studies described CBD in companion animals, specifically dogs. Bartner et al. (2) investigated the pharmacokinetics of CBD in healthy dogs given three formulations of the drug: oral microencapsulate oil bead, oral CBD-infused oil, and CBD-infused transdermal cream. The PK parameters were determined after a single dose administration, as well as long term administration over 6 weeks. This study's results showed that the oral CBD-infused oil formulation had higher systemic exposure than the transdermal cream or microencapsulated oil bead and had a half-life of 3.2 h with a 75-mg dose (~10 mg/kg body weight per day) and 2.1 h with a 150-mg dose (~20 mg/kg body weight per day) (2). At these doses, it appeared that the clearance systems were not saturated, suggesting that evaluating our data using first-order kinetics was appropriate. Another PK study on dogs administered CBD within an enriched oil, reporting a half-life of 4.2 h (1). These studies were the basis for our study design.

More recently, Kleinhenz et al. (7) reported the PK parameters of industrial hemp administered orally in 10-week-old cattle. Specifically, they characterized the PK of cannabidiolic acid (CBDA), the precursor to CBD. The geometric mean maximum concentration of CBDA was 72.7 ng/mL and was found at 11.82 h. The geometric mean half-life was reported at 14.1 h, which was substantially longer than that reported for CBD in dogs (1, 7). Additionally, Kleinhenz et al. only found 2 of 8 calves with detectable CBD in their plasma, likely due to a much lower dose of CBD administered based on calf body weight compared to our work reported here. In that study, calves received an average dose of 0.57 mg/kg (range −0.52–0.63) CBD compared to 5 mg/kg in the current study (7).

The half-life reported here of 23.02 h is increased compared to CBDA following oral administration of industrial hemp in cattle, supporting that a longer persistence of cannaboids in cattle compared to dogs, regardless of the source. In the current study, plasma concentration of CBD remained prominent to the 48 h sampling time, with little decrease in plasma following development of the peak concentration (Figure 1). As a result, the extrapolated portion of the AUC_0−∞_ was slightly more than the 20% that is generally considered as acceptable. This represents one of the weaknesses of the current study. Time points for this study were chosen from canine and equine studies. However, given the extended compound presence found for cattle in this study, an extended study of plasma level would be beneficial to get a more accurate assessment of total drug exposure and to gain further insight into the determination of CBD clearance and a potential withdrawal time following CBD administration to food producing species.

The results from this study were suggestive of a low bioavailability high variability of CBD after oral administration in cattle, which was expected based on the study results of other CBD studies. Canine PK studies have demonstrated highly variable bioavailability of 0–19% (1). Other dog and human PK studies of oral cannabis extracts have also found variable and low bioavailability, reporting that only 5–10% of the drug reaches its target site (9). A review of CBD pharmacology, reports oral absorption of the drug seems to be less bioavailable and more variable when compared to drug absorption *via* inhalation (10). Some of the variability may be explained by the preparation of the CBD in oil as standardized procedures product preparation may not be utilized. While oil extraction is known to more efficacious and less variable than water extraction, the lack of standard preparation could lead to CBD compound differences between company products (11). Within the production companies formulation of CBD compounds can also vary. According to one of the co-authors (JB), the formulation of the CBD compound can be altered which may improve bioavailability, which could serve as the investigatory basis of future work. Variability of CBD may also be affected by the stability of the product. Pacifici et al. (12) found 15–20% of cannabinoids in oil are lost in the first 14 days of storage at 4° C in darkness, but remains stable following the initial decrease for up to a year. Another study in to the stability of CBD, investigated CBD degradation at different temperatures and exposure to air oxygen. The study revealed stability for a period of 180 days for product in oil stored at 25 and 40°C in a closed vial not exposed to air oxygen. Significant degradation, up to 20%, was seen in products with an open vial exposed to air oxygen as early as 90 days (13). Storage conditions may lead to degradation of the CBD within the product, which may lead to some of the variability seen and needs to be taken into consideration when using CBD products.

Calf 6,523 had the lowest C_max_ and AUC and showed a significant deviation in apparent clearance and apparent volume of distribution from the other calves. The individual deviation observed could be attributed to the low bioavailability of CBD administered orally or to a failure of the calf to consume the CBD. We do not believe that the latter is the cause as we closely observed drug administration. Further conclusions cannot be drawn from this observation as there are no plasma bioavailability studies completed in cattle. Future work to determine oral bioavailability of CBD in cattle is required to make definitive conclusions. Additionally, the forestomachs of calves of this age are not active therefore absorption of orally administered drugs may be similar to that of monogastric animals. Given this, investigation into CBD absorption in ruminating cattle is warranted. Drug absorption differences between pre-ruminating and rumination cattle may change the pharmacokinetics of CBD.

The results of this study have implications for CBD as a therapeutic agent in cattle. In past studies it has been found that even with low bioavailability, CBD had been reported to have good clinical responses. A study completed in dogs found CBD given at 2 mg/kg produced a median maximum plasma concentration of 0.102 ug/mL (range −0.61–0.132 ug/mL) and had positive therapeutic implications. In that work, CBD reduced pain scores and increased activity in osteoarthritic dogs compared to dogs that received the placebo (9). Although CBD is not approved in cattle, obtaining similar maximum plasma concentrations in cattle could lead to its use for pain relief, appetite stimulation, and inflammatory modulation. Determination of PK parameters, can guide administration procedures and study design for future investigations into the therapeutic efficacy of CBD in cattle.

This study provides preliminary results describing the fate of the CBD upon oral uptake in cattle. From here, further work can be completed to describe the bioavailability and drug depletion from tissues of CBD. Additionally, investigation into the effectiveness of CBD as a therapeutic agent is required for cattle and other livestock species.

## Data Availability Statement

The original contributions presented in the study are included in the article/supplementary material, further inquiries can be directed to the corresponding author/s.

## Ethics Statement

The animal study was reviewed and approved by Iowa State University's Institutional Animal Use and Care Committee.

## Author Contributions

KM was responsible for acquisition of data, data analysis and interpretation, drafting of the manuscript, and approval of the submitted manuscript. KH was responsible for acquisition of data and manuscript revision. JB was responsible for conception of the study and manuscript revision. PG was responsible for the conception of the study, manuscript writing, pharmacokinetic evaluation, statistical analysis, and revision of the manuscript. All authors contributed to the article and approved the submitted version.

## Funding

We gratefully acknowledge funding received for this research from Panacea Life Sciences.

## Conflict of Interest

The authors declare that the research was conducted in the absence of any commercial or financial relationships that could be construed as a potential conflict of interest.

## Publisher's Note

All claims expressed in this article are solely those of the authors and do not necessarily represent those of their affiliated organizations, or those of the publisher, the editors and the reviewers. Any product that may be evaluated in this article, or claim that may be made by its manufacturer, is not guaranteed or endorsed by the publisher.

## References

[1] Gamble LJ Boesch JM Frye CW Schwark WS Mann S Wolfe L. Pharmacokinetics, safety, and clinical efficacy of cannabidiol treatment in osteoarthritic dogs. Front Vet Sci. (2018) 5:165. 10.3389/fvets.2018.00165 30083539 PMC6065210

[2] Bartner LR McGrath S Rao S Hyatt LK Wittenburg LA. Pharmacokinetics of cannabidiol administered by 3 delivery methods at 2 different dosages to healthy dogs. Can J Vet Res. (2018) 82:178–83. 30026641 PMC6038832

[3] Baumgartner J Dukes L. Equine research on the short-term effects of cannabidiol for the treatment of chronic pain and/or anxiety. Panacea Life Sci. (2020). Available online at: https://panacealife.com/cbd-equine-research/

[4] della Rocca G Di Salvo A. Hemp in veterinary medicine: from feed to drug. Front Vet Sci. (2020) 7:387. 10.3389/fvets.2020.00387 32850997 PMC7399642

[5] Landa L Sulcova A Gbelec P. The use of cannabinoids in animals and therapeutic implications for veterinary medicine: a review. Vet Med. (2016) 61:111–22. 10.17221/8762-VETMED

[6] Deabold KA Schwark WS Wolf L Wakshlag JJ. Single-Dose pharmacokinetics and preliminary safety assessment with use of CBD-rich hemp nutraceutical in healthy dogs and cats. Animals. (2019) 9:10:832. 10.3390/ani9100832 31635105 PMC6826847

[7] Kleinhenz MD Magnin G Lin Z Griffin J Kleinhenz KE Montgomery S. Plasma concentrations of eleven cannabinoids in cattle following oral administration of industrial hemp (*Cannabis sativa*). Sci Rep. (2020) 10:12753. 10.1038/s41598-020-69768-4 32728233 PMC7391639

[8] American Dairy Science Association American American Society of Animal Science Poultry Science Association. Dairy Cattle Facilities and Environment. Guide for the Care and Use of Agriculture Animals in Research and Teaching. 4th edn. Champaign, IL: American Dairy Science Association (2020).

[9] Hartsel JA Boyar K Pham A Silver RJ Makriyannis A. Cannabis in veterinary medicine: cannabinoid therapies for animals. Nutraceut Vet Med. (2019). 10:121–55. 10.1007/978-3-030-04624-8_10

[10] Brunetti P Lo Faro AF Pirani F Berretta P Pacifici R Pichini S. Pharmacology and legal status of cannabidiol. Ann Ist Super Sanita. (2020) 56:285–91. 10.4415/ANN_20_03_06 32959794

[11] Brunetti P Pichini S Pacifici R Busardò FP Del Rio A. Herbal preparations of medical cannabis: a vademecum for prescribing doctors. Medicina. (2020) 56:237. 10.3390/medicina56050237 32429074 PMC7279290

[12] Pacifici R Marchei E Salvatore F Guandalini L Busardò FP Pichini S. Evaluation of long-term stability of cannabinoids in standardized preparations of cannabis flowering tops and cannabis oil by ultra-high-performance liquid chromatography tandem mass spectrometry. Clin Chem Lab Med. (2018) 56:94–6. 10.1515/cclm-2017-0758 29176009

[13] Kosovic E Sykora D Kuchaf M. Stabiliy study of cannabidiol in the form of solid poweder and sunflower oil solution. Pharmaceutics. (2021) 13:412. 10.3390/pharmaceutics13030412 33808893 PMC8003596

